# High‐Isolation Nonreciprocal Sawtooth Grating via Quasi‐Bound States in the Continuum

**DOI:** 10.1002/nap2.70328

**Published:** 2026-10-23

**Authors:** Changqing Zhong, Menghan Liu, Lujun Hong, Yun Shen, Jing Chen, Jiangtao Lei, Tianjing Guo

**Affiliations:** ^1^ Department of Physics School of Physics and Material Science Nanchang University Nanchang China; ^2^ Institute of Space Science and Technology Nanchang University Nanchang China; ^3^ School of Physics Nankai University Tianjin China

**Keywords:** bound states in the continuum (BIC), high isolation, high‐Q metasurfaces, nonreciprocal response

## Abstract

Nonlinear optical nonreciprocity based on high‐Q resonances offers a promising route toward compact integrated photonic devices, yet most reported structures operate at a single resonance and are commonly analyzed using idealized Kerr‐only models. Here, we theoretically investigate a sawtooth‐shaped silicon grating supporting three symmetry‐protected bound states in the continuum (BICs) that evolve into high‐Q quasi‐BICs (QBICs) after in‐plane symmetry breaking. The three resonances exhibit distinct directional coupling asymmetries and consequently different nonlinear nonreciprocal responses. Within the idealized Kerr‐only model, full‐wave simulations predict a maximum NRIR of 2.99, whereas QBIC 2 reaches an isolation exceeding 67 dB at an incident intensity of 12.5 MW/cm^2^. To evaluate realistic parasitic nonlinearities in crystalline silicon, we further incorporate wavelength‐dependent material dispersion, two‐photon absorption, and TPA‐generated free‐carrier absorption and dispersion through a sequential frequency‐domain/time‐domain multiphysics workflow. For QBIC 2, the extended model predicts a representative isolation of 34.8 dB at 1296.13 nm and 12.5 MW/cm^2^. These results clarify how modal symmetry, directional coupling asymmetry, and realistic nonlinear material effects jointly govern multiwavelength nonreciprocal transmission, providing quantitative guidance for future experimental realization.

## Graphical abstract

We demonstrate a silicon sawtooth grating that simultaneously hosts three quasi‐BIC resonances. Leveraging the enhanced local field and directional coupling asymmetry, the device achieves a maximum nonreciprocal intensity ratio (NRIR) of 2.99 and an isolation exceeding 67 dB. This work provides a compact, multiresonant platform for low‐power optical isolators and integrated photonic circuits.

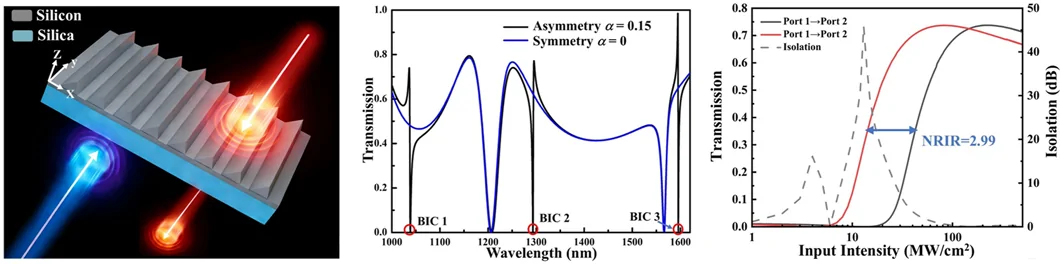

## 1 Introduction

Nonreciprocal optical devices can break through the constraints of Lorentz reciprocity, achieve asymmetric electromagnetic transmission, and make light exhibit drastically different optical responses when propagating in forward and reverse directions [1, 2]. This unique nonreciprocal characteristic makes them indispensable core components in many key fields [3] such as optical communication [4, 5], integrated photonic circuits [6, 7], nonlinear signal processing [8], and quantum optics [9]. According to the constraint conditions of Lorentz reciprocity, reciprocity can be broken through three physical approaches: first, the magneto‐optical effect is used to obtain an asymmetric permittivity tensor to achieve nonreciprocity [10, 11]. However, such devices based on magneto‐optical materials are usually bulky, have complex preparation processes, and require the application of an external static magnetic bias, which seriously hinders their integrated application in miniaturized high‐performance photonic chips [12, 13]. Second, the time‐varying characteristics of permittivity are realized by time‐varying material modulation, and nonreciprocity is achieved by breaking the space–time invariance [14, 15]. Nevertheless, the current research on this method is still relatively scarce. Particularly, nonlinear metasurfaces can induce nonreciprocal characteristics by virtue of their significant electromagnetic nonlinear effects (such as the Kerr effect) [4, 16, 17, 18]. Moreover, such devices have the advantages of simple design and fabrication and no need for external electromagnetic bias, and have become a research hotspot in the field of nonreciprocal devices. However, because of the generally weak nonlinear coefficients of nonlinear materials, nonreciprocal characteristics can be achieved only under relatively high incident light intensity [19]. Therefore, such nonreciprocal devices have a strong dependence on high‐Q optical resonance.

At present, the main optical mechanisms for achieving high‐Q resonance include Fano resonance, guided‐mode resonance, Mie resonance, and bound states in the continuum (BICs) [20, 21, 22, 23]. Among these, BICs have been widely used in metasurface research, especially in nonlinear optics, because of their theoretically infinite Q‐factor and strong electromagnetic field localization capability. A BIC refers to a special optical state that exists within the radiative continuum yet exhibits no radiative leakage [24]. Typically, an ideal BIC possesses an infinitely high Q‐factor and is completely decoupled from the external environment, making it unobservable in practice [24, 25]. However, by introducing perturbations into the system, ideal BICs can be transformed into quasibound states in the continuum (QBICs) with finite high Q‐factors, enabling external excitation and practical applications. BICs can be mainly divided into two categories: symmetry‐protected BICs and accidental BICs [26, 27, 28]. The former arises from the incompatibility between structural symmetry and eigenmodes, whereas the latter arises from destructive interference among radiation channels [29]. The most common type is the symmetry‐protected BIC, which can be converted into a QBIC by breaking internal symmetry (geometric symmetry breaking) or external symmetry (oblique incidence) [30]. The high Q‐factor characteristics of QBICs and their strong electromagnetic field localization capability can significantly enhance the efficiency of nonlinear optical processes [31, 32]. Moreover, the out‐of‐plane geometric asymmetry of the structure enables asymmetric coupling between incident plane waves and the metasurface under forward and reverse propagation directions. By integrating symmetry‐protected BICs with an asymmetric silicon metasurface, the enhanced Kerr effect enables asymmetric optical transmission, providing a compact platform for high‐performance nonreciprocal devices [4, 33].

In this work, we design a sawtooth silicon grating that supports three symmetry‐protected BICs within the infrared band. By displacing the vertex of the unit‐cell triangle away from the center, the in‐plane symmetry is broken, converting the symmetry‐protected BICs into QBICs. The Q‐factors of the three QBIC modes follow an *α* ^−2^ scaling with the asymmetry parameter, whereas their resonance frequencies remain nearly unchanged. Far‐field polarization analysis further reveals that these modes carry a topological charge of |*q*| = 1 at the *Γ* point. Leveraging these three high‐Q resonances within a single structure, the idealized Kerr‐only model predicts distinct nonlinear nonreciprocal responses at the three QBIC channels, including a maximum NRIR of 2.99 and an isolation exceeding 67 dB. These values should be understood as numerical predictions under the Kerr‐only approximation rather than experimentally established device metrics. To assess the robustness of the mechanism against parasitic nonlinearities in crystalline silicon, we additionally develop an extended material model incorporating wavelength‐dependent dispersion, two‐photon absorption (TPA), and TPA‐generated free‐carrier absorption (FCA)/free‐carrier dispersion (FCD). For the QBIC 2 channel responsible for the largest Kerr‐only isolation, the extended model yields a representative isolation of approximately 34.8 dB under the considered pulse conditions. The proposed sawtooth grating therefore provides a compact multiresonant theoretical platform for investigating and designing nonlinear nonreciprocal photonic devices. It should be clarified that all analyses and conclusions in this work rely on full‐wave numerical simulation rather than experimental measurement. We also systematically discuss the practical fabrication and characterization challenges of the proposed device to facilitate future experimental validation.

## 2 Structure Design and Simulation Methods

The designed sawtooth grating structure, illustrated in Figure 1a, consists of periodic silicon triangular prisms atop a silicon slab. For the baseline calculations used to elucidate the symmetry‐controlled QBIC and Kerr‐induced nonreciprocal mechanism, silicon is initially described using a relative permittivity of 12.25 [34, 35, 36], consistent with the idealized Kerr‐only model. To quantitatively assess realistic material effects, we additionally construct an extended crystalline‐silicon model using the wavelength‐dependent complex refractive index *ñ*_Si_(*λ*) = *n*_Si_(*λ*) + i*k*_Si_(*λ*). For wavelengths up to approximately 1.45 μm, both *n*_Si_(*λ*) and *k*_Si_(*λ*) are taken from the self‐consistent optical data of intrinsic crystalline silicon reported by Green [37]. In the longer‐wavelength transparent region, *n*_Si_(*λ*) is supplemented using the evaluated dispersion data of Li [38], whereas the intrinsic extinction coefficient is taken to be negligibly small. The resulting spectral shifts are modest but non‐negligible for the narrow QBIC resonances, although the resonance lineshapes and the symmetry‐protected mechanism remain intact. A refractive index of 1.4 is used for the silica substrate. The unit cell dimensions are as follows: grating period *p*, base length of the triangular prism *L*_1_, prism height *H*_1_, and silicon slab thickness *H*_2_. This configuration is specifically chosen to support high‐quality‐factor (high‐Q) resonances by leveraging the physics of BICs. When the vertex of the triangle lies on the symmetry axis (indicated by the black dashed line in Figure 1b), the grating is in‐plane symmetric, hosting a symmetry‐protected BIC that is entirely confined with infinite *Q*. By shifting the triangle vertex away from the center, the in‐plane symmetry is intentionally broken, converting the ideal BIC into a QBIC with a finite but still high Q‐factor. This symmetry‐breaking mechanism enables strong local field enhancement and also introduces direction‐dependent coupling, both of which are essential for achieving pronounced nonreciprocal response in the nonlinear regime. The asymmetry is quantified by a parameter *α* = *L*_2_/*L*_1_, defined as the ratio of the vertex shift distance from the symmetry center to the base length of the triangle.

**FIGURE 1:**
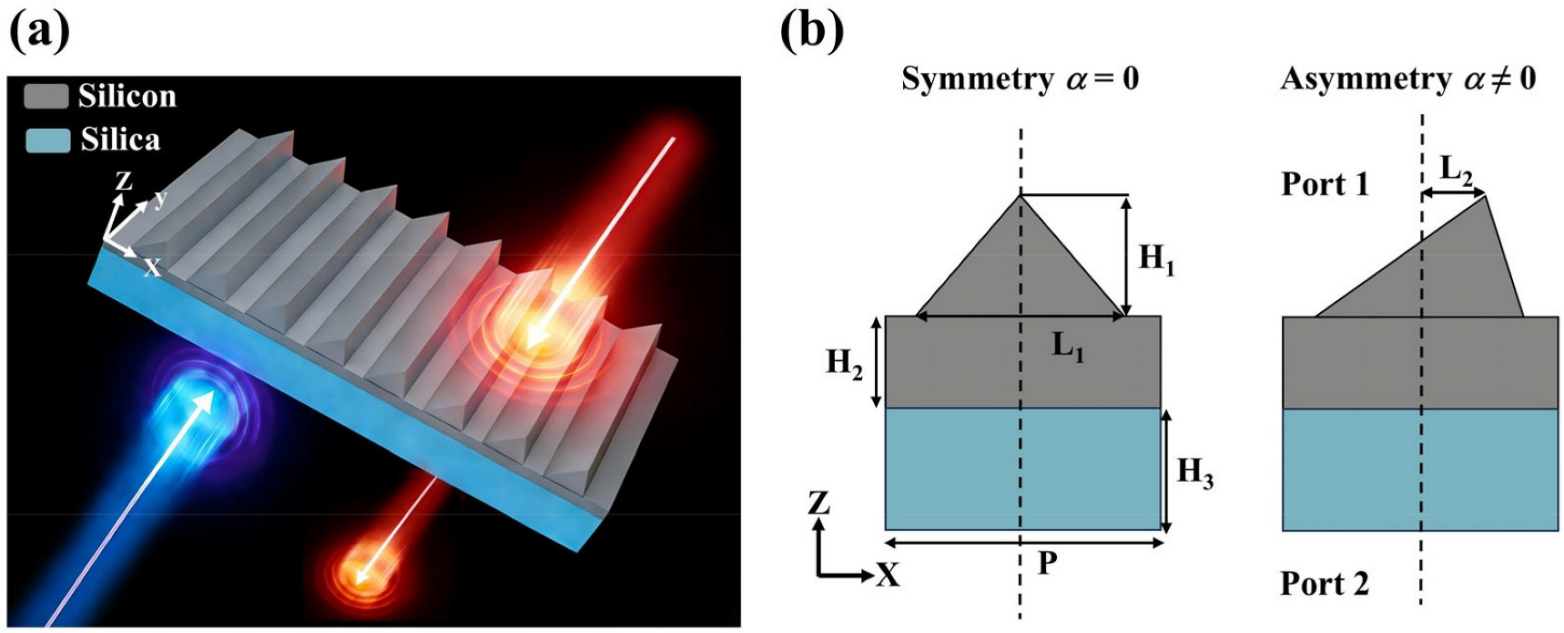
Schematic of the designed metasurface. (a) Perspective view of the metasurface consisting of periodic silicon triangular prisms on a silicon slab and silica substrate. (b) Side view of the unit cell in the x–z plane. The geometric parameters are period *p*, triangle base length *L*_1_, triangle height *H*_1_, silicon slab thickness *H*_2_, and the vertex shift distance *L*_2_.

To analyze the optical properties, a two‐dimensional (2D) model of the grating structure is constructed assuming infinite length along the *y*‐direction, and simulations are performed using the finite element method (FEM) in COMSOL Multiphysics. Floquet–Bloch periodic boundary conditions are applied to the two lateral boundaries along the *x‐*direction, and port boundary conditions are imposed on the upper and lower boundaries along *z*, with a plane wave source having its electric field polarized parallel to the *y*‐axis, corresponding to transverse electric (TE) polarization. We employ a hierarchical mesh employing a 10 nm, 6‐layer boundary mesh at all interfaces, with maximum element sizes of 50 nm for the prism and 150 nm for the slab/substrate, and incorporated field‐gradient adaptive refinement to enhance accuracy. The transmission and reflection spectra are characterized using the *S*‐parameters: *S*_11_ represents the reflection spectrum for incidence from the top port (forward direction), whereas *S*_21_ represents the corresponding transmission spectrum. To model the nonlinear response of silicon, the third‐order nonlinear susceptibility *χ* ^(3)^ = 2.8 × 10^−18^ m^2^/V^2^ [39] is incorporated through the intensity‐dependent permittivity *ε*_nl_ = *ε*_0_ *χ* ^(3)^ *|E*(*r*)*|* ^2^, where *ε*_0_ is the vacuum permittivity and *|E*(*r*)*|* is the local electric field amplitude [40]. Directly solving this full nonlinear expression leads to severe convergence failure for ultrahigh‐Q modes, because extreme field confinement induces sharp, discontinuous spatial variation of Kerr permittivity. We address this issue via the nonlinearity ramping continuation method recommended by COMSOL official numerical guidance. A global dimensionless parameter *g* ∈ [0,1] is introduced to smooth the silicon permittivity, and the nonlinear part is described as Δ*ε*_Kerr_(*g*) = *gε*_0_ χ^(3)^∣*E*∣^2^. When *g* = 0, silicon is fully linear to provide a stable baseline solution. We sweep *g* from 0 to 1 via an auxiliary parametric sweep with an initial step of 0.1. The built‐in continuation algorithm reuses converged field profiles of smaller *g* as initial guesses for larger values, and automatically backtracks to finer intermediate steps if divergence occurs. This gradual activation of nonlinearity avoids abrupt permittivity jumps and spurious spectral shifts of narrow Fano resonances. When *g* = 1, the formula recovers the complete third‐order Kerr nonlinear model. This approach enables a self‐consistent evaluation of the nonlinear field distribution and, consequently, the intensity‐dependent nonreciprocal transmission performance.

To evaluate parasitic nonlinear effects in crystalline silicon, we extend the Kerr‐only model by incorporating the wavelength‐dependent complex refractive index [37, 38], wavelength‐dependent TPA [39, 41], and TPA‐generated free carriers [42]. The carrier dynamics are solved using a time‐dependent domain ODEs and DAEs interface in COMSOL, and the resulting carrier distribution is subsequently introduced into a second frequency‐domain optical calculation through a Drude‐type complex‐permittivity correction [43], thereby including both FCD and FCA. The complete calculation follows a sequential frequency‐domain optical → time‐dependent carrier → frequency‐domain optical workflow. Further details of the material parameters and numerical implementation are provided in Supporting Information S1: Section S5, Figure S5, and Table S1, and the quantitative QBIC 2 reassessment is presented in Supporting Information S1: Section S6, Figure S6, and Table S2 [44].

## 3 Results and Discussion

### 3.1 Analysis of BIC^s^ Formation

We first analyze the generation of QBICs by calculating the transmission spectra of the sawtooth grating under both symmetric and asymmetric configurations, as shown in Figure 2a. The optimized unit cell parameters are as follows: grating period *p* = 500 nm, base length of the triangular prism *L*_1_ = 400 nm, prism height *H*_1_ = 300 nm, and silicon slab thickness *H*_2_ = 320 nm. The thickness of the silica substrate is not specified, as it does not affect the transmission spectrum of the grating (see Supporting Information S1: Figure S1 [44]). For the unperturbed case with asymmetry parameter *α* = 0, the structure retains perfect in‐plane mirror symmetry and supports symmetry‐protected BICs, indicated by the red circles in Figure 2a. These modes remain entirely confined within the structure with no coupling to free‐space radiation channels [30]. By displacing the vertex of the sawtooth grating, the in‐plane symmetry is intentionally broken. Under this asymmetric condition (*α* = 0.15), the previously trapped BIC modes acquire a pathway for radiative loss, and the BIC response transitions into QBIC resonances with finite but still high Q‐factors. This transition manifests as three ultranarrow asymmetric Fano‐shaped transmission dips in the calculated spectra (black curve in Figure 2a), observed at wavelengths of 1038.8, 1293.3, and 1595.6 nm. The absence of these resonance features in the symmetric case confirms that they originate from symmetry‐protected BICs, whose transformation into QBICs is directly induced by the controlled symmetry breaking.

**FIGURE 2:**
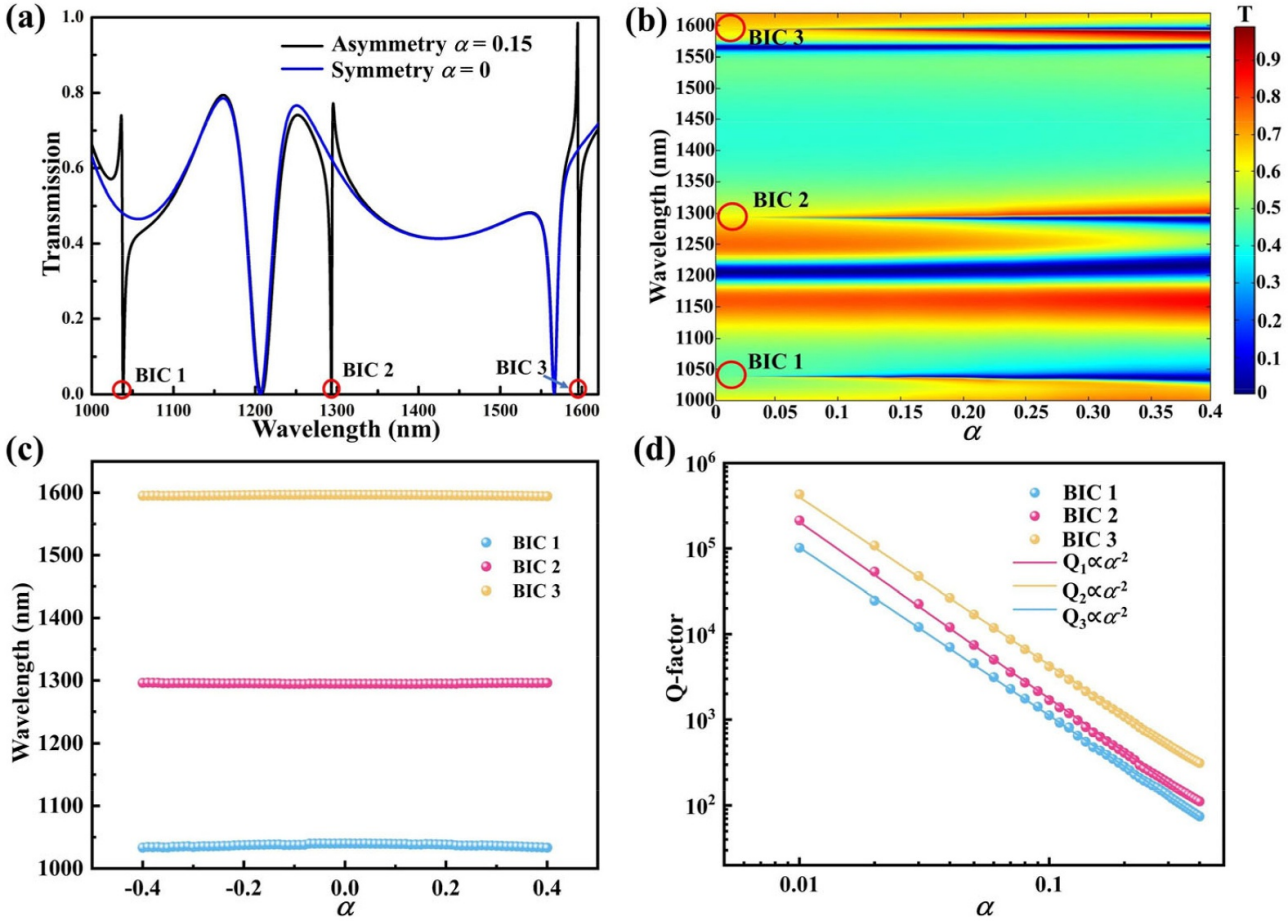
(a) Transmission spectra of the sawtooth grating under symmetric conditions (blue curve) and with asymmetry parameter *α* = 0.15 (black curve). The symmetry‐protected BICs are indicated by red circles. (b) Two‐dimensional color map showing the transmission spectra as functions of incident wavelength and asymmetry parameter *α*. The ideal BIC states in the limit *α* → 0 are marked by red circles. (c) Resonance wavelengths of the three BIC modes as a function of *α*. The negative values of *α* indicate that the triangle vertex is shifted to the left relative to the symmetry center. (d) Q‐factors of the three BIC modes as a function of *α*.

To further elucidate the formation of BICs and their dependence on the asymmetry parameter *α*, we present a two‐dimensional color map in Figure 2b, which illustrates the evolution of the transmission spectrum as a function of both incident wavelength and *α*. As *α* approaches zero, the resonance linewidth gradually narrows, and the resonance features disappear, indicating that the QBIC modes progressively converge to pure BICs with vanishing radiation loss. With increasing *α*, the resonance linewidth broadens monotonically, reflecting the enhanced coupling to free‐space radiation channels. Notably, the resonance wavelengths of the three BIC modes remain nearly constant across the entire range of *α* (Figure 2c), underscoring the robustness of their spectral positions against symmetry breaking. This behavior further confirms the distinct identity of these modes as symmetry‐protected BICs in the unperturbed limit. We further quantify the resonant properties by performing eigenfrequency simulations to calculate the Q‐factors of the QBIC modes as a function of *α*. The Q‐factor is determined by *Q = ω/2γ*, where *ω* denotes the real part of the eigenfrequency and *γ* represents its imaginary part [23, 45]. The results, presented in Figure 2d, clearly show that the Q‐factors of the three QBIC modes follow an *α* ^−2^ dependence, consistent with findings reported in previous studies [30]. For the specific asymmetry parameter *α* = 0.15, the Q‐factors of the three modes are 480, 714, and 1878, respectively. These values, combined with the frequency stability demonstrated in Figure 2c, confirm that the proposed structure supports high‐Q resonances with robust spectral positioning, well suited for nonlinear nonreciprocal applications.

We analyze the mode characteristics of the BICs by first examining the band structure of the grating under perfectly in‐plane symmetric conditions (*α* = 0) and then tracking the evolution of each mode with the wave vector *k*_*x*_, as shown in Figure 3a,b. The results reveal that the Q‐factors of the three modes tend to infinity at the point and gradually decrease as they deviate from it. Further analysis of the polarization vectors and Q‐factors in momentum space (see Supporting Information S1: Figure S2 [44]) indicates that the polarization vectors exhibit a vortex‐like distribution around the *Γ* point, and the Q‐factors diverge precisely at this point. Quantitative evaluation confirms that the absolute values of the topological charges for all three BICs at the *Γ* point satisfy ∣*q*∣ = 1. These features are characteristic of symmetry‐protected BICs in momentum space [46, 47]. Figure 3c presents the electric field distributions in the x–z plane for the three BIC modes at the *Γ* point under in‐plane symmetric conditions. The electric fields are strongly confined within the silicon triangular prism and the silicon slab regions, with negligible radiation into the surrounding background. This confinement, arising from structural symmetry protection, results in an infinite Q‐factor, consistent with the behavior shown in Figure 3b. However, their distribution features reveal significant differences in mode order: BIC 1 exhibits three pairs of odd–even mode distributions along the vertical direction, BIC 2 exhibits two pairs, and BIC 3 exhibits only one pair. This variation in mode order directly influences how each mode couples to the radiation continuum when in‐plane symmetry is broken. Specifically, higher‐order modes possess more complex spatial field distributions, which lead to stronger overlap with free‐space radiation channels under the same asymmetry parameter *α*. Consequently, these modes experience larger radiative losses, manifesting as broader resonance linewidths and lower peak transmittance. In contrast, the lowest‐order mode (BIC 3) exhibits the weakest coupling to radiation, thereby retaining a higher Q‐factor and yielding the highest peak transmittance. This trend is fully consistent with the transmission spectra shown in Figure 2a. As we have demonstrated above, the three modes observed in our structure are identified as symmetry‐protected BICs. This identification is supported by three characteristic signatures: (i) the resonance linewidth vanishes at the symmetry point and broadens monotonically with increasing asymmetry parameter *α* (Figure 2b); (ii) the Q‐factors follow the universal scaling *Q*∝*α* ^−2^ (Figure 2d); and (iii) the Q‐factors diverge at the *Γ* point and decrease rapidly with *k*_*x*_ (Figure 3b). These features collectively confirm that the modes are protected by in‐plane mirror symmetry and transition to QBICs only when this symmetry is intentionally broken.

**FIGURE 3:**
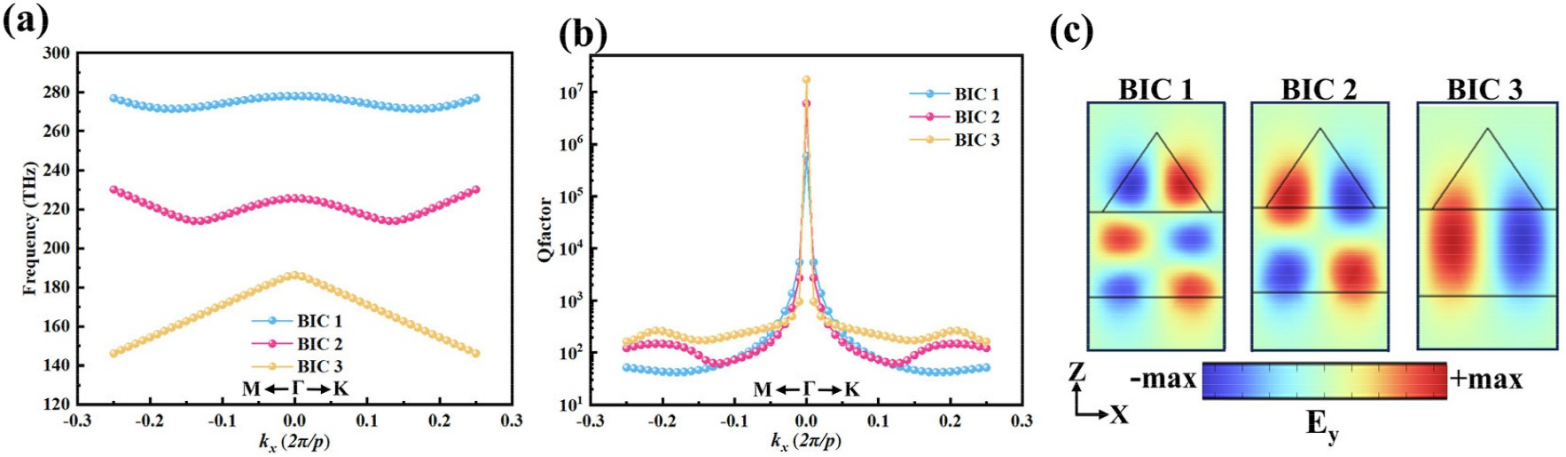
(a) Band structure diagram of BICs. (b) Evolution of BICs with wave vector *k*_*x*_. (c) Electric field distributions of BIC modes in the x–z plane.

### 3.2 CMT Fitting

For a two‐port system, if its frequency response is governed by a complex frequency *ω*_*i*_ = *ω* + *iγ*, the full‐wave behavior can be described using a set of first‐order differential equations. Temporal coupled‐mode theory (CMT) has been widely employed in previous studies and has been shown to accurately reproduce the results of full‐wave electromagnetic simulations [48, 49]. The structure proposed in this work can also be analyzed using CMT. For a linear, time‐invariant two‐port resonator system, the transmission spectrum can be described by the following equation [2]:

(1) $$T\left(\omega \right)={\left|\frac{t_{0}\left(\omega_{0}-\omega \right)\pm \sqrt{4\gamma_{1}\gamma_{2}-t_{0}^{2}{\left(\gamma_{1}+\gamma_{2}\right)}^{2}}-it_{0}\gamma_{n}}{i\left(\omega_{0}-\omega \right)+\gamma_{1}+\gamma_{2}}\right|}^{2}$$

Here, *ω*_0_ is the resonance frequency, and *t*_0_ denotes the transmission coefficient of the background spectrum. The parameters *γ*_*n*_, *γ*_*1*_, and *γ*_*2*_ represent nonradiative loss, the radiative decay rate at Port 1, and the radiative decay rate at Port 2, respectively. These decay rates are related to the asymmetric coupling coefficient *κ* via *κ = γ*_*1*_ */γ*_*2*_, which governs the asymmetry of the Fano resonance lineshape. Figure 4 presents the CMT‐fitted transmission curves for the three QBICs at an asymmetry parameter *α* = 0.15. The fitting results show excellent agreement with the full‐wave simulations performed using the steady‐state solver in COMSOL Multiphysics. The distinct maximum transmittances of the three QBICs can be well explained by CMT, as they originate from differences in their asymmetric coupling coefficients *κ*.

**FIGURE 4:**
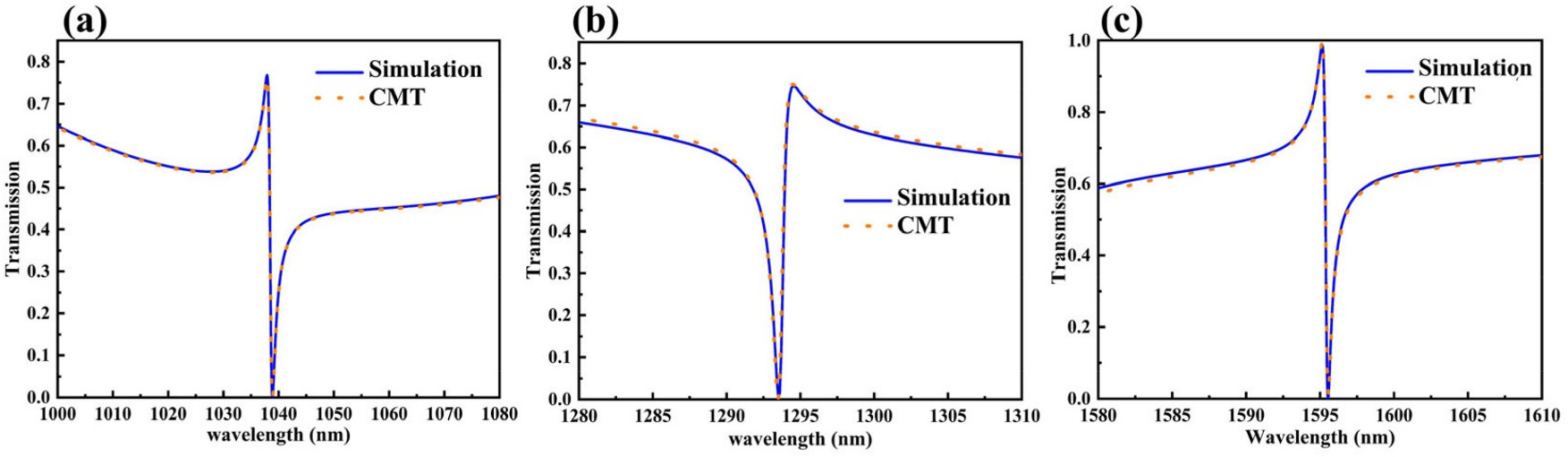
Comparison between CMT‐fitted curves (orange dotted lines) and simulated transmission spectra (blue solid lines): (a) BIC 1, (b) BIC 2, and (c) BIC 3.

The nonreciprocal intensity range is intrinsically linked to the electromagnetic asymmetry in the linear regime. Specifically, when equal power is injected from Port 1 and Port 2, the ratio of the resulting field intensities *|E*_*i*_ *|* ^2^(*i* = 1, 2) at the same point satisfies *κ = |E*_2_ *|* ^2^ */|E*_1_ *|* ^2^ [16]. The electric field distributions of the three QBICs at their resonance wavelengths under illumination from Port 1 and Port 2 are shown in Supporting Information S1: Figure S3 [44], where the corresponding intensity ratios closely adhere to the above relationship. Owing to their varying degrees of electromagnetic asymmetry, the three QBIC modes are expected to exhibit distinctly different nonreciprocal responses.

### 3.3 Nonreciprocal Characteristics

Having established in the preceding linear analysis that the sawtooth grating supports high‐Q resonances with asymmetric port coupling, we now introduce optical nonlinearity to investigate the resulting nonreciprocal behavior. Based on the analysis presented in the previous sections, the three QBIC modes—owing to their distinct electromagnetic asymmetries—are expected to exhibit markedly different nonreciprocal responses. The fact that our structure simultaneously supports three QBIC resonances provides a convenient platform to systematically explore the relationship between mode characteristics and nonreciprocal performance. Moreover, although all three QBIC resonances can appear in the broadband transmission spectrum, each resonance can be selectively addressed using a narrowband excitation at its corresponding wavelength, offering both multiwavelength and single‐mode operational flexibility.

The nonlinear results presented in Figure 5a–i are obtained using the idealized Kerr‐only model and are primarily used to elucidate the intrinsic relationship among field enhancement, asymmetric port coupling, and nonlinear resonance detuning. We simulate the forward and reverse transmission spectra of the three QBICs under varying incident intensities using the stationary solver in COMSOL Multiphysics, with results shown in Figure 5a–c. Here, “1 → 2” denotes transmission from Port 1 to Port 2 (forward direction), whereas “2 → 1” denotes transmission from Port 2 to Port 1 (reverse direction). Under linear conditions, the forward and reverse transmissions are identical, satisfying the Lorentz reciprocity theorem [50]. Upon introducing nonlinearity, both transmission spectra exhibit redshifts, which become more pronounced with increasing incident intensity. This behavior arises from the intensity‐dependent permittivity variation, which scales quadratically with the local electric field. Moreover, the nonlinear permittivity *ε*_nl_ amplifies the inherent asymmetry in port coupling, leading to an increasing difference in frequency shifts between the two propagation directions as the incident power increases—that is, a higher isolation. Comparing Figure 5a–c, at the same incident intensity, the relative redshift for QBIC 3 is significantly larger than those for QBIC 1 and QBIC 2. This is attributed to the substantially higher Q‐factor of QBIC 3 (1878) compared with QBIC 1 (480) and QBIC 2 (714). A higher Q‐factor indicates stronger field localization, leading to a larger intensity‐dependent permittivity change and consequently a more pronounced redshift even at relatively low incident intensities. This trend is consistent with the field enhancement factors at resonance for each QBIC under linear conditions (see Supporting Information S1: Figure S3 [44]). These results underscore the critical role of both electromagnetic asymmetry and Q‐factor in tailoring the nonreciprocal performance of multiwavelength devices.

**FIGURE 5:**
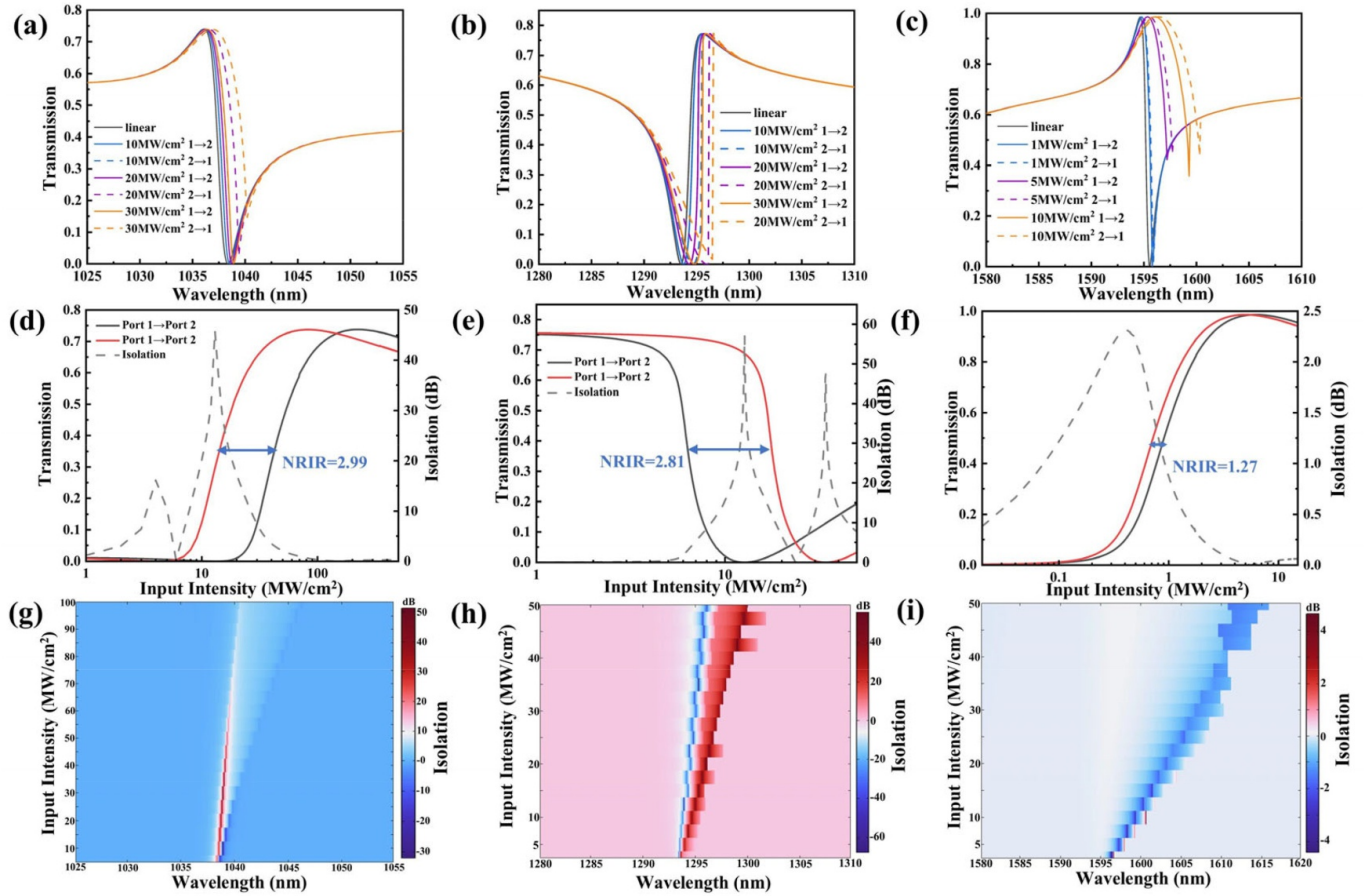
(a–c) Nonreciprocal characteristics of the three QBIC resonances. Forward‐ and reverse‐transmission curves for (a) QBIC 1, (b) QBIC 2, and (c) QBIC 3 under different incident optical intensities. “1 → 2” indicates transmission from Port 1 to Port 2 (forward direction), and “2 → 1” indicates transmission from Port 2 to Port 1 (reverse direction). (d–f) The NRIR and isolation for (d) QBIC 1, (e) QBIC 2, and (f) QBIC 3 at the selected operating wavelength. (g–i) Simulated isolation level as a function of incident wavelength and optical intensity for (g) QBIC 1, (h) QBIC 2, and (i) QBIC 3. The results shown in this figure correspond to the idealized Kerr‐only nonlinear model; the influence of wavelength‐dependent TPA and free‐carrier effects is evaluated separately using the extended crystalline‐silicon model described in Section 2 and Supporting Information S1: Sections S5 and S6.

The NRIR provides a specific quantification of the nonreciprocity strength, calculated as NRIR = max(*I*_1_, *I*_2_)/min(*I*_1_, *I*_2_), where *I*_1_ and *I*_2_ represent the excitation intensities at the two ports that yield the same transmittance. We evaluate the NRIR by sweeping the incident intensity at the QBIC resonances and computing the forward and reverse transmittance, with the results shown in Figure 5d–f. For QBIC 1 and QBIC 3, the incident wavelengths are 1038.8 and 1595.6 nm, respectively, corresponding to the wavelengths of minimum transmittance in the linear regime. QBIC 2 exhibits an asymmetric Fano lineshape; therefore, to maximize the nonlinear contrast under intensity sweeping, the incident wavelength is set at the peak transmittance (1294.5 nm) rather than the dip, consistent with the approach in previous work [51]. The calculated NRIR values for QBIC 1, QBIC 2, and QBIC 3 are 2.99, 2.81, and 1.27, respectively. Notably, although these three QBICs arise from the same geometric structure, they yield distinct NRIR values. This is because each QBIC corresponds to a distinct resonant mode with a unique near‐field distribution and symmetry. According to CMT, the NRIR is determined by the ratio of the decay rates to the two ports; that is, NRIR = max(*γ*_*1*_ */γ*_*2*_, *γ*_*2*_ */γ*_*1*_). Different QBIC modes exhibit different *γ*_*1*_ and *γ*_*2*_ because of their distinct coupling efficiencies to the two ports, leading to varying NRIR values even within the same physical structure. It has been previously established that the maximum transmittance *T*_max_ and NRIR satisfy the constraint *T*_max_ ≤ 4 × NRIR/(NRIR^2^ + 1) [2]. Our structure, which supports multiple QBICs, directly validates this relationship, confirming the predicted trade‐off: a higher *T*_max_ yields a lower NRIR. Specifically, QBIC 3 exhibits a very low NRIR of only 1.27 because of its high *T*_max_ of 0.98, whereas QBIC 1 and QBIC 2 both have maximum transmittance values below 0.8, resulting in substantially larger NRIR values.

We further consider nonreciprocal transmission, that is, isolation, which characterizes the transmission selectivity of the device when excited from different ports. The isolation is calculated as *NT* = 10 × log_10_[max(*T*_*1*_,*T*_*2*_)/min(*T*_*1*_,*T*_*2*_)] [52], where *T*_*1*_ and *T*_2_ denote the transmittance for forward and backward propagation under the same pump power. The first two QBIC resonances achieve strong isolation alongside high NRIR values, whereas QBIC 3 exhibits low values for both metrics, as illustrated in Figure 5d–f. To comprehensively evaluate the isolation performance, we calculate the isolation under varying incident intensities and wavelengths, with the results presented in Figure 5g–i. Within the idealized Kerr‐only model, QBIC 1 reaches a numerically predicted maximum isolation of approximately 50 dB at an incident intensity of around 30 MW/cm^2^, whereas QBIC 2 reaches approximately 67 dB at around 12.5 MW/cm^2^. These values characterize the intrinsic Kerr‐dominated response of the model and should not be interpreted as experimentally established performance benchmarks. Notably, although QBIC 1 exhibits a higher NRIR, QBIC 2 attains a larger isolation at a lower pump power. This is primarily attributed to the significantly higher Q‐factor of QBIC 2, which enhances the local electric field and thus induces stronger nonlinear effects.

However, a higher *T*_max_ and a higher Q‐factor do not necessarily guarantee superior isolation performance. Despite having a *T*_max_ close to unity and a Q‐factor substantially higher than those of the other two resonances, QBIC 3 achieves a maximum isolation of only 4 dB. This is because *T*_max_ is strongly correlated with the asymmetric coupling coefficient *κ* [53, 54]. A *T*_max_ approaching unity implies that the two ports are nearly symmetrically coupled (i.e., *γ*_1_ ≈ *γ*_2_), leading to almost identical nonlinearity‐induced frequency shifts in the forward and reverse directions, even when the absolute frequency shift is large because of the high Q‐factor. Consequently, the nonreciprocal effect is strongly suppressed. In summary, isolation is governed by both the asymmetric coupling coefficient and *T*_max_. Under an appropriate *T*_max_, resonances with higher Q‐factors are more favorable for achieving high isolation. Previous studies have shown that out‐of‐plane asymmetry can be enhanced by modifying the height difference at the base of the grating, thereby tailoring the port electromagnetic asymmetry to improve isolation performance [16, 35]. However, silicon gratings are highly sensitive to variations in these geometric parameters, which can lead to significant shifts in the resonance frequency. Consequently, constrained by the physical mechanism of nonlinear nonreciprocal isolators, optimizing one performance metric (e.g., isolation) often comes at the expense of another (e.g., NRIR or insertion loss), necessitating a trade‐off in practical device design. To guide this trade‐off, we systematically map the nonreciprocal performance of QBIC 1 as a function of the out‐of‐plane parameters *H*_1_ and *H*_2_. The Pareto‐optimal analysis, detailed in Figure S4 in the Supplementary Material [44], reveals that increasing *H*_1_ and *H*_2_ enhances NRIR at the expense of reduced *T*_max_. For an isolator requiring isolation > 40 dB and insertion loss < 1 dB (*T*_max_ > 0.794), the optimal parameters are *H*_1_ ≈ 250 nm and *H*_2_ ∈ [310, 320] nm, yielding NRIR ≈ 2.35–2.45 and isolation ≈ 42.5–42.8 dB.

To quantitatively explain the physical origin of the distinct nonreciprocal performance among the three QBIC modes, we extract the radiative decay rates *γ*_1_ and *γ*_2_ to the two ports from the CMT fits for each mode at *α* = 0.15. The results are summarized in Table 1. In the ideal two‐port CMT description, the coupling asymmetry *γ*_1_/*γ*_2_ sets the characteristic scale of the NRIR, expressed as max(*γ*_1_/*γ*_2_, *γ*_2_/*γ*_1_). For QBIC 3, the fundamental mode exhibits a symmetric field distribution with nearly equal intensities at the top and bottom interfaces, as shown in Figure 3c, yielding *γ*_1_/*γ*_2_ ≈ 1.1 and thus a low NRIR of 1.27. This explains why QBIC 3, despite its highest Q‐factor of 1878, achieves only 4 dB isolation—a high Q‐factor is necessary but insufficient without strong coupling asymmetry. In contrast, QBIC 1 and QBIC 2 are higher‐order modes with complex multilobe field patterns and substantial intensity imbalance between the two interfaces. Their uneven field overlap with the two ports produces large *γ*_1_/*γ*_2_ values of 3.9 and 3.0, respectively, which amplify the directional nonlinear resonance shifts and lead to much higher NRIR values of 2.99 and 2.81. This analysis explicitly links the modal field symmetries in Figure 3c to the observed nonreciprocal performance, revealing that high isolation requires both a high Q‐factor and a strong coupling asymmetry. In the ideal two‐port CMT description, the coupling‐rate ratio (*γ*_*1*_ */γ*_*2*_) sets the characteristic scale of the NRIR. The NRIR values extracted from the finite‐intensity nonlinear simulations follow the same trend but need not coincide exactly with the coupling ratios obtained from the linear CMT fits.

**TABLE 1:** CMT‐extracted coupling asymmetry and nonreciprocal response.

QBIC mode	*γ*_*1*_ */γ*_*2*_	Q‐factor	NRIR	Isolation (dB, Kerr‐only)
QBIC 1	3.9	480	2.99	50
QBIC 2	3.0	714	2.81	67
QBIC 3	1.1	1878	1.27	4

The isolation values listed in Table 1 are obtained from the idealized Kerr‐only model and are used to compare the intrinsic nonlinear responses of the three QBIC modes. For QBIC 2, the extended crystalline‐silicon model including wavelength‐dependent dispersion, TPA, FCA, and FCD gives a representative isolation of approximately 34.8 dB at 1296.13 nm and 12.5 MW/cm^2^.

To evaluate how realistic parasitic nonlinearities modify the largest isolation predicted by the Kerr‐only model, we further reassess QBIC 2 using the extended crystalline‐silicon model including wavelength‐dependent dispersion, TPA, TPA‐generated carriers, FCA, and FCD. Because the carrier‐induced refractive‐index change shifts the narrow nonlinear resonance, wavelength continuation followed by fine spectral refinement is performed around the shifted backward‐transmission minimum. At an incident peak intensity of 12.5 MW/cm^2^, the minimum is located near 1296.13 nm. At this wavelength, the extended model gives *T*_F_ = 0.49555, *R*_F_ = 0.49885, and *A*_F_ = 0.00560 for forward incidence, whereas *T*_B_ = 1.631 × 10^−4^, *R*_B_ = 0.95797, and *A*_B_ = 0.04187 for backward incidence. The corresponding isolation is 10log_10_(*T*_F_/*T*_B_) = 34.83 dB. Further details are provided in Supporting Information S1: Section S6, Figure S6, and Table S2 [44]. Thus, realistic carrier effects substantially modify the optimum isolation predicted by the Kerr‐only model, although the forward‐to‐backward transmission ratio remains approximately 3.0 × 10^3^.

To contextualize the numerically predicted characteristics of the proposed design, Table 2 compares representative nonlinear nonreciprocal structures reported in the literature. We emphasize that the values listed for the present work in the main comparison columns are obtained from the idealized Kerr‐only model and therefore do not constitute experimentally demonstrated benchmarks. The principal distinction of the present design is the simultaneous support of three spectrally separated QBIC channels within a single compact structure. For QBIC 2, the extended material calculation incorporating crystalline‐silicon dispersion, TPA, and free‐carrier effects gives a representative isolation of approximately 34.8 dB at 12.5 MW/cm^2^. Table 2 is therefore intended to provide contextual comparison rather than to claim experimentally demonstrated superiority. The coexistence of multiple high‐Q QBIC modes originating from identical geometric symmetry breaking provides a straightforward, compact route toward multiwavelength integrated optical isolators, which is a distinct advantage of our scheme compared with prior art.

**TABLE 2:** Performance comparison of recently reported nonlinear nonreciprocal metasurfaces based on BICs/high‐Q resonances.

References	Platform	Q‐factor	Pump intensity (MW/cm^2^)	NRIR	Isolation (dB)	Operating band
2025 [55]	Asymmetric groove Si cylinder metasurface	3852.6	0.28	1.63	34.28	Single (2.3204 THz)
2025 [19]	Si photonic crystal slab	—	0.04	7.1	∼5^a^	Single (∼1.83 μm)
2023 [34]	Si cylinders with eccentric holes	—	40	2.2	∼16^b^	Single (∼1180 nm)
2025 [51]	Plate‐hole tetramer metasurface	< 1000^c^	∼1	3.59	> 15	Single (∼1.6 μm)
2026 [56]	Si waveguide with periodic grooves	24,395, 29,830	0.212, 0.429	1.4, 3.7	71.16, 80.98	Dual (1.59 μm, 1.91 μm)
2025 [57]	THz metamaterial	< 1000	60	1.77	23.97	Single (0.854 THz)
This work	Sawtooth Si grating	480, 714, 1878	Mode‐dependent^d^	2.99, 2.81, 1.27	50, 67, 4	Three (1038.8, 1293.3, 1595.6 nm)

^a^ The isolation value is calculated using the definition 10 × log_10_(*T*_max_/*T*_min_), where *T*_max_ and *T*_min_ are the maximum and minimum transmittance read from Figure 4a in Ref. [19].

^b^ The isolation value is calculated using the definition 10 × log_10_(*T*_max_/*T*_min_), where *T*_max_ and *T*_min_ are the maximum and minimum transmittance read from Figure 4b in Ref. [34].

^c^ The data are taken at *H* = 180 nm, where the maximum NRIR is achieved in Figure 5d in Ref. [51].

^d^ The maximum Kerr‐only isolation values are obtained at mode‐dependent incident intensities. In particular, QBIC 1 reaches approximately 50 dB at 30 MW/cm^2^, whereas QBIC 2 reaches approximately 67 dB at 12.5 MW/cm^2^.

### 3.4 Practical Challenges for Experimental Realization

Although the proposed sawtooth grating offers a promising theoretical platform for multiwavelength nonreciprocal transmission, several practical challenges must be addressed before experimental demonstration can be realized. We discuss these challenges from four critical aspects. The first aspect is fabrication precision. The symmetry‐breaking parameter *α* = *L*_2_/*L*_1_ = 0.15 corresponds to a 60 nm vertex offset relative to the 400 nm triangle base. To maintain high Q‐factors, electron‐beam lithography (EBL) and reactive‐ion etching (RIE) must control pattern positioning errors and linewidth fluctuations within 10 nm [58, 59]. Moreover, fabricating the tilted sawtooth sidewalls with ideal profiles is extremely challenging. Imperfect etching angles simultaneously break both in‐plane and out‐of‐plane symmetry, degrading the directional coupling asymmetry and hence the nonreciprocal performance. Uniform thickness control of the 320 nm silicon slab and 300 nm prism across the entire sample further imposes strict requirements on SOI wafer quality and etching uniformity [60]. Any periodic defects or dimensional deviations introduce extra radiative loss and sharply reduce resonance Q‐factors [58]. The second aspect is optical excitation conditions. The QBIC resonances are optimized for normal TE‐polarized incidence. Angular or polarization deviations can modify the radiative coupling, broaden or shift the resonances, and reduce the attainable Q‐factors. Slight oblique angles or polarization impurities destroy the vortex polarization distribution at the *Γ* point, suppress field localization, and eliminate the high‐Q QBIC resonances [61]. The three resonant wavelengths span from 1038.8 to 1595.6 nm with ultranarrow linewidths, requiring a high‐resolution near‐infrared spectroscopic system to resolve the Fano dips—a configuration not routinely available in standard laboratory setups. The third aspect is nonlinear characterization. The target nonreciprocal response originates from silicon's third‐order Kerr nonlinearity [39]. For the QBIC 2 operating point considered here, the numerical model uses a peak incident intensity of 12.5 MW/cm^2^. In real samples, inevitable reflection, scattering, and substrate absorption demand substantially higher input peak power, relying on high‐power pulsed near‐infrared lasers [62]. Moreover, thermo‐optic effects induced by residual absorption overlap with the electronic Kerr nonlinearity, introducing spurious spectral redshifts that contaminate the intrinsic nonreciprocal signal [3].

Beyond thermal effects, TPA and TPA‐generated carriers can also modify the nonlinear resonance. As quantified by the extended model described in Section 2 and Supporting Information S1: Sections S5 and S6 [44], inclusion of TPA, FCA, and FCD shifts the QBIC 2 operating point to approximately 1296.13 nm and reduces the representative isolation from the idealized Kerr‐only value of 67 dB to approximately 34.8 dB at 12.5 MW/cm^2^. This result confirms that free‐carrier effects are quantitatively important, although pronounced direction‐dependent transmission remains. A fully symmetric bidirectional optical path is mandatory to compare forward (Port 1 → 2) and backward (Port 2 → 1) transmission. Any unbalanced loss between the two directions will produce false nonreciprocal contrast. The very low backward transmission predicted near the nonlinear operating point places stringent requirements on detector noise floor, dynamic range, and bidirectional calibration. Low‐noise detection techniques such as lock‐in amplification may therefore be desirable in future experimental characterization. The last aspect is multiwavelength measurement. Unlike conventional single‐BIC nonreciprocal metasurfaces, our structure supports three independent QBIC resonances across a 550 nm bandwidth. Broadband optical components (lenses, polarizers, and beam splitters) cannot maintain identical coupling efficiency over such a wide range, leading to distorted measured NRIR and isolation values for different modes. Each resonance requires separate scanning of incident power and wavelength to reach optimal performance, making the experimental characterization extremely time‐consuming. In addition, the inherent trade‐off between peak transmittance and NRIR leaves a narrow parameter window for balancing insertion loss and isolation during device optimization. These considerations define the principal experimental constraints associated with the proposed design and provide practical targets for future fabrication and characterization. The present numerical analysis therefore serves primarily as a theoretical framework for evaluating modal coupling, nonlinear operating conditions, fabrication tolerances, and the trade‐off between transmission and nonreciprocal contrast.

## 4 Conclusion

In conclusion, we have designed a sawtooth silicon grating that supports three symmetry‐protected BICs within the infrared band. By displacing the vertex of the triangular prism, we intentionally break the in‐plane symmetry, converting these ideal BICs into QBICs with high but finite Q‐factors. Band structure analysis reveals that the three QBIC modes exhibit robust resonance frequencies that remain nearly unchanged with varying asymmetry, whereas their Q‐factors follow an *α* ^−2^ scaling. Momentum‐space analysis further confirms that these modes carry a topological charge of |*q*| = 1 at the *Γ* point, characteristic of symmetry‐protected BICs. Using CMT, we quantitatively describe the NRIR as NRIR = max(*γ*_*1*_ */γ*_*2*_, *γ*_*2*_ */γ*_*1*_), where *γ*_1_ and *γ*_2_ are the decay rates to the two ports, linking the observed NRIR variations among the three QBICs to their distinct near‐field distributions and coupling asymmetries. Within the idealized Kerr‐only model, the simulations predict a maximum NRIR of 2.99 and a maximum isolation exceeding 67 dB. We further develop an extended crystalline‐silicon model including wavelength‐dependent dispersion, TPA, and TPA‐generated FCA/FCD. For QBIC 2 at 12.5 MW/cm^2^, this model predicts a representative isolation of approximately 34.8 dB at 1296.13 nm, demonstrating that realistic carrier effects substantially modify the quantitative performance, although they preserve pronounced nonlinear nonreciprocity. The reported nonreciprocal performance metrics should therefore be understood as numerical predictions rather than experimentally established device benchmarks. We also systematically analyze the inherent trade‐off between peak transmittance and NRIR, revealing that a moderate transmittance is essential for achieving strong nonreciprocal effects. Through comprehensive numerical modeling, this work establishes a compact multiresonant theoretical platform and provides quantitative design guidance for future experimental studies of nonlinear nonreciprocal photonic devices.

## Author Contributions

**Changqing Zhong:** conceptualization, methodology, software, investigation, validation, writing – original draft. **Menghan Liu:** software, investigation. **Lujun Hong:** data curation, investigation, validation, visualization. **Yun Shen:** data curation, investigation, visualization. **Jing Chen:** validation, formal analysis, supervision, resources. **Jiangtao Lei:** methodology, funding acquisition, visualization, writing – review and editing, project administration. **Tianjing Guo:** writing – review and editing, project administration, supervision, resources, methodology, conceptualization.

## Supporting information


Supporting Information S1

## Data Availability

The data that support the findings of this study are available from the corresponding author upon reasonable request.
