# Supplementary material for: High‐Isolation Nonreciprocal Sawtooth Grating via Quasi‐Bound States in the Continuum
Source: Nanophotonics. 2026 Oct 23;15(20):e70328. doi: 10.1002/nap2.70328 (PMC13691531; doi:10.1002/nap2.70328)
Supplement: Supplementary file 1 [file NAP2-15-e70328-s001.docx]

**Supplemental Material**

**High-Isolation Nonreciprocal Sawtooth Grating via Quasi-Bound States in the Continuum**

*Changqing Zhong^1^, Menghan Liu^1^, Lujun Hong^2^, Yun Shen^1,2^, Jing Chen^3^, Jiangtao Lei^2,*^, Tianjing Guo^2, †^*

^1^Department of Physics, School of Physics and Material Science, Nanchang University, Nanchang 330031, China

^2^Institute of Space Science and Technology, Nanchang University, Nanchang 330031, China

^3^School of Physics, Nankai University, Tianjin 300071, China

**S1 Robustness to Silica Substrate Thickness**

Figure S1 presents the transmission spectra of the grating under an asymmetric condition (*α* =0.15) for varying substrate thicknesses. The silica thickness was increased from 500 nm to 2000 nm in 250 nm steps. Evidently, the transmission spectra of the grating remain nearly unchanged with varying silica thickness, and the resonance peaks of the QBICs show no observable shift. This demonstrates the strong robustness of our designed grating to variations in substrate thickness.

**Figure S1**. Transmission spectra of the grating under asymmetric conditions (α=0.15) for different substrate thicknesses.

**S2 Far-field polarization and Q-factor map of the sawtooth silicon grating in momentum space**

Under in-plane symmetric conditions (*α* =0) for the silicon grating, we calculated the far-field polarization and Q factors of the BICs by scanning *k_x_* and *k_y_*, as shown in Figure S2. Clearly, their polarization vectors form a vortex (whirlpool) distribution around the Γ point, while the Q factors diverge (approach infinity) at the Γ point. A characteristic feature of such vortices is their topological charge. Here, the topological charge (q) carried by a BIC is defined as[1, 2]:

 (S1)

It describes how many times the polarization vector rotates around the BIC. Here,is the angle of the polarization vector, where. The field *u_k_* is chosen as a smooth function of k, so *ϕ(k)* is differentiable along a path in *k*. *C* is a simple closed path in k-space that encircles the BIC counterclockwise. Since the polarization vector must return to itself after completing a closed loop, the total angle change must be an integer multiple of 2π, and thus q must be an integer. According to the calculation from Eq. (S1), the polarization vector rotates by an angle of ±2π along a closed loop in k-space that encloses the BICs (+2π corresponds to a topological charge q=1, while -2π corresponds to q=-1), indicating that the absolute value of their topological charge is |q|=1.

**Figure S2**. Far-field polarization in momentum space for (a) BIC 1, (b) BIC 2, and (c) BIC 3. Resonant radiation Q-factor maps in momentum space for (d) BIC 1, (e) BIC 2, and (f) BIC 3.

**S3 Electric field intensity distributions under forward and backward incidence at the resonant wavelength for the asymmetric grating (*α* =0.15) in the linear regime.**

We calculated the electric field enhancement distributions for the asymmetric silicon grating at the QBIC resonance wavelengths under both forward transmission (incident from Port 1, output from Port 2) and backward transmission (incident from Port 2, output from Port 1). Since the Q-factor of QBIC 1 is lower than that of QBIC 2, which in turn is lower than that of QBIC 3, QBIC1 exhibits the smallest field enhancement. Under forward transmission, its maximum electric field enhancement is 8.4-fold, while under backward transmission, it reaches 15.2-fold. Its electromagnetic asymmetry parameter *𝜅=|E₂|²/|E₁|²* is 3.2, which closely matches its NRIR (2.99). Next, for QBIC2, the maximum electric field enhancement under forward transmission is 10.3-fold, and under backward transmission, it is 17.9-fold. Its electromagnetic asymmetry parameter *𝜅* is 3.02, also closely matching its NRIR (2.81). Finally, for QBIC 3, the maximum electric field enhancement under forward transmission is 22.8-fold, and under backward transmission, it is 25.8-fold. Its electromagnetic asymmetry parameter *𝜅* is 1.28, while its NRIR is 1.27. This clearly demonstrates a strong correlation between the NRIR of the nonreciprocal device and the electromagnetic asymmetry coefficient. The nonreciprocal grating we designed also follows this trend.

**Figure S3**. Electric field enhancement distributions for (a) QBIC 1, (b) QBIC 2, and (c) QBIC 3 for forward and reverse incident light at the resonant wavelength.

**S4 Quantitative Design Guidelines**

To establish practical design guidelines, we perform a systematic parameter sweep over H₁ ∈ [250, 300] nm and H₂ ∈ [300, 350] nm, with other parameters fixed at α = 0.15, L₁ = 400 nm, and P = 500 nm. As discussed in the main text, the in‑plane parameters primarily determine the Q‑factor via symmetry‑protected BIC conversion, whereas the out‑of‑plane parameters H₁ and H₂ directly control the port coupling asymmetry γ₁/γ₂, which is the key physical origin of the nonreciprocal response. Therefore, the parameter sweep focuses on H₁ and H₂ to provide actionable design guidance for tuning the nonreciprocal performance.

For each parameter set, we take **QBIC 1** as a representative example and compute its NRIR, isolation, and T*_max_*. **Figures S4(a–c)** show the color maps of the three metrics. NRIR increases monotonically with both H₁ and H₂ (from ~2.4 to ~5.0), isolation peaks at ~55 dB in the mid‑range (H₁ ≈ 270 nm, H₂ ≈ 340 nm), and T*_max_* decreases monotonically (from ~0.83 to ~0.56). **Figure S4(d)** presents the Pareto‑optimal plot. All simulated points lie below the theoretical bound T*_max_* = 4×NRIR/(NRIR^2^+1). The green circle marks the target design for isolation > 40 dB and insertion loss (IL) < 1 dB (T*_max_* > 0.794). It can be seen that the parameter combinations that strictly satisfy the requirements for QBIC 1 are (H₁, H₂) = (250, 310) nm and (250, 320) nm. The recommended parameter window is therefore H₁ ≈ 250 nm and H₂ ∈ [310, 320] nm. For applications that tolerate slightly higher insertion loss (~1.0–1.1 dB), (H₁, H₂) = (260, 320) nm and (270, 320) nm provide higher NRIR values of 2.57 and 2.65, respectively, while maintaining isolation above 40 dB.

**Figure S4**. (a–c) Color maps of NRIR, isolation, and *T*_max_ in the H₁–H₂ parameter space. (d) Pareto front of *T_max_* versus NRIR with isolation color‑coding. The dashed curve shows the theoretical bound *T_max_* = 4×NRIR/(NRIR²+1). The green circle indicates the target design space (isolation > 40 dB, *T_max_* > 0.794).

**S5 Extended crystalline-silicon material model and carrier-dynamics simulation**

To assess the influence of parasitic nonlinearities beyond the idealized Kerr-only model used for the baseline mechanism analysis, we construct an extended crystalline-silicon model that explicitly includes wavelength-dependent linear dispersion, two-photon absorption (TPA), TPA-generated carrier dynamics, free-carrier dispersion (FCD), and free-carrier absorption (FCA). The calculation is implemented in COMSOL Multiphysics using a sequential frequency-domain optical → time-dependent carrier → frequency-domain optical workflow. The overall numerical workflow is summarized in Figure S5, and the material and carrier parameters used in the model are listed in Table S1.

**S5.1 Wavelength-dependent linear and nonlinear material parameters**

The wavelength-dependent complex refractive index of crystalline silicon is written as

$\tilde{n}_{\mathrm{Si}}\left( \lambda\right)=n_{\mathrm{Si}}\left( \lambda\right)+i k_{\mathrm{Si}}\left( \lambda\right),$	(S2)

where *n*_Si_(λ) and *k*_Si_(λ) are the real refractive index and extinction coefficient, respectively. For wavelengths up to approximately 1.45 μm, the intrinsic-silicon optical constants are taken from the self-consistent tabulation of Green [3]. In the transparent longer-wavelength range, the refractive-index dispersion is supplemented using the evaluated data of Li [4]. The intrinsic linear extinction is very small in this transparent region compared with the nonlinear absorption considered below. The same third-order Kerr susceptibility, χ^(3)^ = 2.8×10⁻¹⁸ m²/V², as used in the baseline model of the main text is retained.

The local optical intensity used to evaluate nonlinear absorption is calculated from the frequency-domain electric field according to

$I_{\mathrm{loc}}=\frac{1}{2}n_{\mathrm{Si}}\varepsilon_{0}c\left| E \right|^{2}$. (S3)

The wavelength-dependent TPA coefficient β_TPA_(λ) is implemented as an interpolation function based on bulk crystalline-silicon measurements by Bristow et al. over 850–2200 nm [5] and the telecommunication-band measurements of Dinu et al. [6]. Representative interpolated values are approximately 2.0 cm/GW near QBIC1, approximately 0.8 cm/GW near QBIC 2, and approximately 0.75 cm/GW near QBIC 3. The TPA-induced absorption and extinction coefficients are evaluated locally as

$\alpha_{\mathrm{TPA}}=\beta_{\mathrm{TPA}}\left( \lambda\right)I_{\mathrm{loc}}, k_{\mathrm{TPA}}=\frac{\alpha_{\mathrm{TPA}}\lambda}{4\pi}.$	(S4)

Thus, the TPA loss follows the spatially nonuniform QBIC near-field enhancement rather than being introduced as a uniform post-processing attenuation.

**S5.2 Time-dependent TPA-generated carrier dynamics**

To represent the high peak intensity required for nonlinear operation while avoiding a continuous-wave thermal-loading assumption, the local frequency-domain intensity is multiplied by a Gaussian temporal envelope:

$I\left( \mathbf{r},t \right)=I_{\mathrm{loc}}\left( \mathbf{r} \right)\exp\left[ -4\ln2\left( \frac{t-t_{0}}{t_{p}} \right)^{2} \right],$	(S5)

where *t*_p_ = 10 ps is the intensity full width at half maximum (FWHM) and *t*_0_ = 40 ps is the pulse center. The TPA-generated electron–hole pair generation rate is then calculated as [7]

$G_{\mathrm{TPA}}\left( \mathbf{r},t \right)=\frac{\beta_{\mathrm{TPA}}\left( \lambda\right)I^{2}\left( \mathbf{r},t \right)}{2\hbar\omega}.$	(S6)

and the carrier density is obtained from the rate equation [7]:

$\frac{\partial N_{\mathrm{fc}}}{\partial t}=G_{\mathrm{TPA}}-\frac{N_{\mathrm{fc}}}{\tau_{\mathrm{fc}}}.$	(S7)

with the initial condition

$N_{\mathrm{fc}}\left( t\mathbf{=}0 \right)=0$	(S8)

A representative carrier lifetime τ_fc_ = 1 ns is used. Effective carrier lifetimes on the order of 1 ns have been reported for submicron silicon-on-insulator waveguides [9]; however, τ_fc_ is treated here as a representative modeling parameter because the experimentally realized lifetime depends on surface recombination, defect density, geometry, and carrier extraction conditions. In COMSOL, Eq. (S7) is solved only in the silicon domains using the Domain ODEs and DAEs interface. The optical electric field is not re-solved during this carrier step; instead, the converged field from the preceding frequency-domain Kerr+TPA calculation provides the spatial source term in Eq. (S6). COMSOL uses physics-controlled internal time stepping, and the carrier distribution at the pulse center (*t*=*t*_0_=40 ps) is retained for the final optical calculation.

The 10 ps pulse duration is longer than the photon lifetime of the QBIC2 resonance. Using *Q*≈714 gives τ_ph_=*Q*/ω_0_≈0.49 ps. Therefore, the intracavity optical field can be treated as quasi-steady during carrier buildup, supporting the sequential optical–carrier treatment adopted here.

**S5.3 Free-carrier dispersion and absorption**

The carrier density obtained from Eq. (S7) is subsequently introduced into a second frequency-domain electromagnetic calculation through a Drude-type correction to the complex silicon permittivity [8]:

$\Delta\varepsilon_{\mathrm{FC}}=-\frac{\omega_{p,e}^{2}}{\omega\left( \omega+i\gamma_{e} \right)}-\frac{\omega_{p,h}^{2}}{\omega\left( \omega+i\gamma_{h} \right)}$	(S9)

where the electron and hole plasma frequencies and scattering rates are

$\omega_{p,j}^{2}=\frac{N_{\mathrm{fc}}e^{2}}{\varepsilon_{0}m_{j,eff}}, \gamma_{j}=\frac{e}{m_{j,eff}\mu_{j}}, j\in\{e,h\}.$	(S10)

The conductivity effective masses and room-temperature carrier mobilities used in the model are *m*_e,eff_=0.26*m*_0_, *m*_h,eff_=0.39*m*_0_, μ_e_=0.145 m²V⁻¹s⁻¹, and μ_h_=0.045 m²V⁻¹s⁻¹, consistent with standard crystalline-silicon transport parameters [10]. The real and imaginary parts of Δε_FC_ generate FCD and FCA, respectively. In the weak-perturbation form used to update the optical constants,

$\Delta n_{\mathrm{FC}}\approx\frac{\mathrm{Re}\left( \Delta\varepsilon_{\mathrm{FC}} \right)}{2n_{\mathrm{Kerr}}}, k_{\mathrm{FCA}}\approx\frac{\mathrm{Im}\left( \Delta\varepsilon_{\mathrm{FC}} \right)}{2n_{\mathrm{Kerr}}},$	(S11)

where *n*_Kerr_ denotes the refractive index after the Kerr correction has been applied. The total complex refractive index used in the final optical calculation is therefore

$\tilde{n}_{\mathrm{tot}}=n_{\mathrm{Kerr}}+\Delta n_{\mathrm{FC}}+i\left( k_{\mathrm{lin}}+k_{\mathrm{TPA}}+k_{\mathrm{FCA}} \right)$	(S12)

where *k*_lin_, *k*_TPA_, and *k*_FCA_ denote the intrinsic linear, TPA-induced, and free-carrier extinction coefficients, respectively.

**Table S1.** Material and carrier parameters used in the extended crystalline-silicon model.

| Parameter | Value / implementation | Reference / basis |
| --- | --- | --- |
| *n*_Si_(λ) | Wavelength-dependent interpolation | Green [3] up to 1.45 μm;  Li [4] for longer wavelengths |
| *k*_Si_(λ) | Wavelength-dependent interpolation | Green [3] up to 1.45 μm; negligible in the longer-wavelength transparent region |
| χ^(3)^ | 2.8×10⁻¹⁸ m²/V² | Baseline Kerr model |
| β_TPA_, QBIC 1 region | ≈ 2.0 cm/GW | Bristow et al. [5] |
| β_TPA_, QBIC 2 region | ≈ 0.8 cm/GW | Bristow et al. [5]; Dinu et al. [6] |
| β_TPA_, QBIC 3 region | ≈ 0.75 cm/GW (interpolated) | Bristow et al. [5] |
| Pulse FWHM, *t*_p_ | 10 ps | Simulation setting |
| Pulse center, *t*_0_ | 40 ps | Simulation setting |
| Carrier lifetime, τ_fc_ | 1 ns | Representative value; Ref. [9] |
| *m*_e,eff_, *m*_h,eff_ | 0.26*m*_0_, 0.39*m*_0_ | Standard Si parameters [10] |
| μ_e_, μ_h_ | 0.145, 0.045 m²V⁻¹s⁻¹ | Standard Si parameters [10] |
| Carrier continuation, *s*_FC_ | 0–1 in steps of 0.05 | Simulation setting |

**S5.4 Sequential multiphysics workflow and numerical continuation**

The complete workflow is illustrated in Figure S5. In Study I, the wavelength-dependent linear dispersion, Kerr response, and TPA are included in a stationary frequency-domain optical calculation. In Study II, the resulting local field drives the time-dependent carrier equation (S7). In Study III, the carrier distribution at *t* = 40 ps is imported as a variable not solved for into a second stationary electromagnetic calculation. To avoid numerical instability caused by the abrupt carrier-induced shift of the high-Q resonance, the free-carrier contribution is activated using a continuation parameter *s*_FC_ from 0 to 1 in increments of 0.05. Forward and backward incidence are calculated in separate models with identical material parameters, mesh, pulse parameters, and continuation settings; the directional contrast therefore originates from the different nonlinear field buildup and carrier generation associated with the asymmetric port coupling.

**Figure S5.** Sequential multiphysics workflow used for the extended crystalline-silicon calculation. Study I evaluates the wavelength-dependent Kerr+TPA optical field in the frequency domain; Study II calculates the TPA-generated carrier density using the time-dependent rate equation; and Study III imports the carrier distribution at the pulse center into the full optical calculation including FCD and FCA. The free-carrier contribution is activated by continuation from s_FC_ = 0 to 1.

**S6 Quantitative Influence of TPA and Free-Carrier Effects on QBIC 2**

QBIC 2 is selected for detailed reassessment because it produces the largest isolation in the idealized Kerr-only model. Since FCD shifts the narrow nonlinear resonance, the extended-material response cannot be evaluated reliably only at the original Kerr-only operating wavelength. We therefore perform wavelength continuation followed by fine spectral refinement around the shifted backward-transmission minimum. The resulting wavelength dependence and free-carrier continuation are shown in Figure S6, while the final forward and backward power coefficients are summarized in Table S2.

**Table S2.** Extended-material QBIC2 response at 1296.13 nm and 12.5 MW/cm².

| Direction | T | R | A |
| --- | --- | --- | --- |
| Forward | 0.49555 | 0.49885 | 0.00560 |
| Backward | 1.631×10⁻⁴ | 0.95797 | 0.04187 |

For the port definitions used in both directional calculation files, the transmittance, reflectance, and absorption are evaluated from the S-parameters as

$T=\left| S_{21} \right|^{2}, R=\left| S_{11} \right|^{2}, A=1-T-R.$	(S13)

At an incident peak intensity of 12.5 MW/cm², the backward full-model transmittances at 1296.12, 1296.13, 1296.14, and 1296.15 nm are 1.87×10⁻⁴, 1.63×10⁻⁴, 1.68×10⁻⁴, and 2.02×10⁻⁴, respectively [Figure S6(a)]. The minimum is therefore located near 1296.13 nm. At this wavelength, the final (*s*_FC_ = 1) forward and backward responses are listed in Table S2. The isolation is defined as

$Isolation=10\log_{10}\left( \frac{T_{F}}{T_{B}} \right)$	(S14)

Using *T*_F_ = 0.49555 and *T*_B_ = 1.631×10⁻⁴ gives an isolation of 34.83 dB. The corresponding forward-to-backward transmission ratio is approximately 3.0×10³. Figure S6(b) further shows the evolution of the forward and backward transmittances as the free-carrier contribution is gradually activated. The backward response changes non-monotonically with *s*_FC_, demonstrating that the free-carrier correction is not equivalent to a simple additional absorption loss; FCD also shifts and reshapes the high-Q nonlinear resonance.

**Figure S6.** Extended-material QBIC2 response at an incident peak intensity of 12.5 MW/cm². (a) Fine wavelength refinement of the backward full-model transmittance, showing the minimum near 1296.13 nm. (b) Forward and backward transmittances at 1296.13 nm as the free-carrier contribution is continued from s_FC_ = 0 to 1. The pronounced change in the backward branch reflects both carrier-induced resonance detuning and nonlinear absorption.

The extended model therefore reduces the representative QBIC 2 isolation from the 67 dB optimum predicted by the idealized Kerr-only model to 34.83 dB under the pulse and carrier-lifetime parameters considered here. This value is not claimed as a globally optimized or universal experimental limit, because the exact result depends on carrier lifetime, pulse duration, fabrication-induced loss, thermal effects, and other device-specific parameters. Nevertheless, the backward transmission remains at the 10^-4^ level, and pronounced nonlinear nonreciprocity is preserved after TPA, FCA, and FCD are included.

**References:**

[1] B. Zhen, C. W. Hsu, L. Lu, A. D. Stone, M. Soljačić, Physical Review Letters, 113 (2014) 257401.

[2] R.-L. Chern, J.-C. Chang, H.-C. Yang, Scientific Reports, 13 (2023) 14139.

[3] M. A. Green, Solar Energy Materials and Solar Cells, 92 (2008) 1305–1310.

[4] H. H. Li, Journal of Physical and Chemical Reference Data, 9 (1980) 561–658.

[5] A. D. Bristow, N. Rotenberg, H. M. van Driel, Applied Physics Letters, 90 (2007) 191104.

[6] M. Dinu, F. Quochi, H. Garcia, Applied Physics Letters, 82 (2003) 2954–2956.

[7] Q. Lin, O. J. Painter, G. P. Agrawal, Optics Express, 15 (2007) 16604–16644.

[8] R. A. Soref, B. R. Bennett, IEEE Journal of Quantum Electronics, 23 (1987) 123–129.

[9] D. Dimitropoulos, R. Jhaveri, R. Claps, J. C. S. Woo, B. Jalali, Applied Physics Letters, 86 (2005) 071115.

[10] S. M. Sze, K. K. Ng, Physics of Semiconductor Devices, 3rd ed., Wiley-Interscience, Hoboken, NJ (2007).
